# Neuroprotective Efficacy of Methylene Blue in Ischemic Stroke: An MRI Study

**DOI:** 10.1371/journal.pone.0079833

**Published:** 2013-11-21

**Authors:** Qiang Shen, Fang Du, Shiliang Huang, Pavel Rodriguez, Lora Talley Watts, Timothy Q. Duong

**Affiliations:** 1 Research Imaging Institute, University of Texas Health Science Center at San Antonio, San Antonio, Texas, United States of America; 2 Department of Ophthalmology, University of Texas Health Science Center at San Antonio, San Antonio, Texas, United States of America; 3 Department of Radiology, University of Texas Health Science Center at San Antonio, San Antonio, Texas, United States of America; 4 Department of Cellular Structural Biology, University of Texas Health Science Center at San Antonio, San Antonio, Texas, United States of America; 5 Department of Neurology, University of Texas Health Science Center at San Antonio, San Antonio, Texas, United States of America; 6 South Texas Veterans Health Care System, Department of Veterans Affairs, San Antonio, Texas, United States of America.; National University of Singapore, Singapore

## Abstract

Methylene blue (MB) has unique energy-enhancing and antioxidant properties and is FDA-approved drug to treat methemoglobinemia and cyanide poisoning. This study evaluated the efficacy of MB to treat ischemic stroke in rats using longitudinal MRI and behavioral measures. Rats were subjected to 60-minute middle-cerebral-artery occlusion. In a randomized double-blinded design, vehicle or MB was administered after reperfusion. The initial lesion volumes at 30 minutes post-ischemia were not significantly different between the two groups (P = 0.92). The final infarct volumes two days after stroke increased in the vehicle group but decreased in the MB group, yielding a 30% difference in infarct volume (P = 0.03). Tracking tissue fate on a pixel-by-pixel basis showed that MB salvaged more initial core pixels compared to controls (22±3% versus 11±3%, P = 0.03), and more mismatch pixels compared to controls (83±3% versus 61±8%, P = 0.02). This study demonstrates MB treatment minimizes ischemic brain injury and improves functional outcomes.

## Introduction

Despite the tremendous efforts invested in ischemic stroke research, the ability to minimize infarct volume and neurological deficit remains extremely limited [1]. Recombinant tissue plasminogen activator (rtPA) – the only drug clinically approved to treat ischemic stroke – is unfortunately limited to only a small fraction of acute stroke patients because rtPA has a short treatment window and risk of hemorrhagic transformation. While many neuroprotective treatments have been explored, none have been translated into the clinics, making the need to develop novel treatment strategies for stroke ever more urgent.

Methylene blue USP (MB) is a FDA-grandfathered drug currently used to treat methemoglobinemia, and cyanide poisoning in humans (see review [2], [3]). MB's auto-oxidizing property acts as an electron cycler [3] that allows MB to redirect electrons to the mitochondrial electron transport chain (in the absence of oxygen), thereby sustaining or enhancing ATP production and promoting cell survival. In bypassing complex I–III activity to generate ATP, MB reduces reactive oxygen species production from the mitochondrial electron transport chain species [4], [5], which has the potential to minimize ischemic and reperfusion injury. MB has recently been shown to reduce behavioral impairments in animal models of Parkinson's disease [3], Alzheimer's disease [6], [7], and reduce cerebral infarct volume by histology [5].

The goal of this study was to test the hypothesis that MB treatment reduces infarct volume and neurologic deficit following cerebral ischemia. The efficacy of MB was evaluated in the treatment of ischemic stroke on a transient (60 mins) focal cerebral ischemia in rats. Quantitative mulitparametric MRI were used to longitudinally monitor and track ischemic progression on a pixel-by-pixel basis. Neurologic status was also evaluated and compared with imaging findings.

## Methods

### Animal Preparation

**Figure 1:**
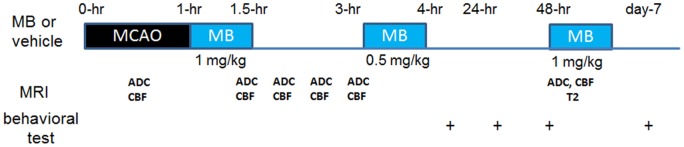
Schematic drawings of experimental design.

All experimental procedures were approved by the Institutional Animal Care and Use Committees of the University of Texas Health Science Center San Antonio. A randomized and double-blinded experimental design was utilized. Transient (60-min) focal cerebral ischemia of the right hemisphere was induced by intraluminal filament middle-cerebral-artery occlusion (MCAO) in male Sprague-Dawley rats (250–350 g, n = 16) under 2% isoflurane [8]. Rats were mechanically ventilated and secured in the supine position using a MRI-compatible rat stereotaxic headset. Anesthesia was reduced to 1.2% to 1.3% isoflurane and maintained during MRI study. Imaging was performed during occlusion and animal was slid out on the rail to withdraw the filament, while the animal was in the holder at 60 minutes after occlusion. Either vehicle or MB (1 mg/kg) was infused over 30 mins intravenously through the tail vein starting immediately after reperfusion, again at 3-hr post-occlusion (0.5 mg/kg), and on day-2 (1 mg/kg). Four rats were excluded due to the absence of mismatch or incomplete occlusion determined by MRI at 30 mins after MCAO. End-tidal CO_2_, rectal temperature, heart rate and arterial oxygenation saturation were recorded and maintained within normal physiological ranges during MRI [8]. Standard neurological tests, utilizing the 6-point NINDS scale (0 =  no deficit, 1 =  failure to extend left forepaw fully, 2 =  circling to the left, 3 =  falling to the left, 4 =  no spontaneous walking with a depressed level of consciousness, and 5 =  dead) [9], were performed on day 0 after the animal recovered from anesthesia, and on days 2 and 7. The experimental design is summarized in **Figure 1**.

### MRI Experiments

Imaging was performed on a Bruker Biospec 7T/40 cm scanner with a 76G/cm BGA12S gradient insert (Billerica, MA) using a custom-made surface coil for brain imaging and a neck coil for perfusion labeling [8]. Cerebral blood flow (CBF) was measured using continuous arterial spin labeling with gradient echo-planar imaging. Continuous arterial spin-labeling used a 2.7-s square radiofrequency pulse to the labeling coil. Post-labeling delay was 250 ms. Apparent diffusion coefficient (ADC) was measured using spin-echo diffusion-weighted echo-planar imaging with gradients separately applied along the x, y, or z direction. Two b values of 4 and 1,200s/mm^2^ were used. Other MRI parameters were: single shot, matrix = 96×96 (reconstructed to 128×128), field of view  = 25.6×25.6 mm, seven 1.5 mm thick slices, 90° flip angle, repetition time  = 3 s, echo time  = 10.2 ms for CBF and 30 ms for ADC. ADC and CBF maps were acquired at 30, 90, 120, 150, and 180 mins post-occlusion and on day-2 (48-hr). T2 maps were also acquired on day-2 using fast spin echo with four effective echo times (25, 40, 75 and 120 ms), echo train length 8, and 8 signal averages.

### Data Analysis

Images from each rat at different time points were co-registered [8], [10]. ADC, CBF and T2 maps were calculated [8], [10]. Three tissue types (normal, perfusion-diffusion mismatch and ischemic core) were defined based on 30-min (first MRI time point) ADC and CBF maps using the auto-clustering ISODATA method [10]. The volume of the ischemic core was used as the initial lesion volume. Final infarct volume was defined using day-2 T2 maps using the threshold of the mean T2 value from the normal hemisphere plus two times the standard deviation. To correct for the effects of edema, a corrected infarct volume was calculated by the following formula: corrected infarct volume  =  infarct volume – (right hemisphere volume – left hemisphere volume) [11]. The ADC, CBF values and fate of initial ischemic core and mismatch (determined using ISODATA method at 30-min post-occlusion) were tracked over time. The lesion volumes determined using day-0 ADC maps and day-2 T2 maps were compared between MB-treated and control groups.

### Statistical analysis

Data were reported as mean ± SEM. Paired t-test was used for comparison between initial lesion volume and final infarct volume, and the unpaired t-test was utilized for comparisons between treated and control groups. P<0.05 was taken to be statistically significant.

## Results

**Figure 2:**
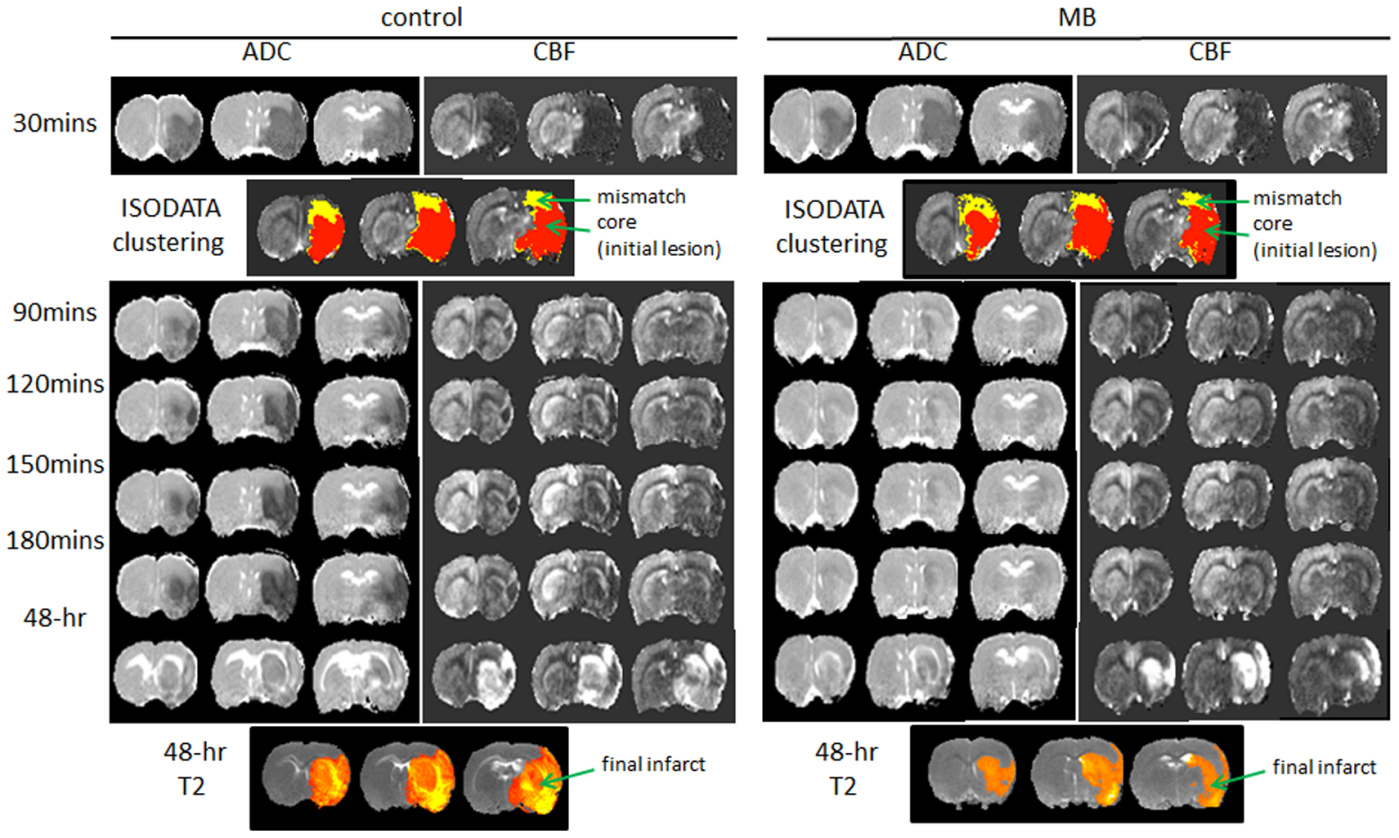
(A) ADC, CBF maps at 30, 90, 120, 150, 180 mins and 48 hrs post-occlusion, ISODATA clustering results (determined using 30-min ADC and CBF) and T2 maps 48 hrs post-occlusion (day-2) of vehicle- and MB-treated rats subjected to 60-mins MCAO are shown. ISODATA determined core (red) tissue volume was used as the initial lesion volume. Final infarct volume was determined based on day-2 T2 maps using the threshold method.

**Figure 3:**
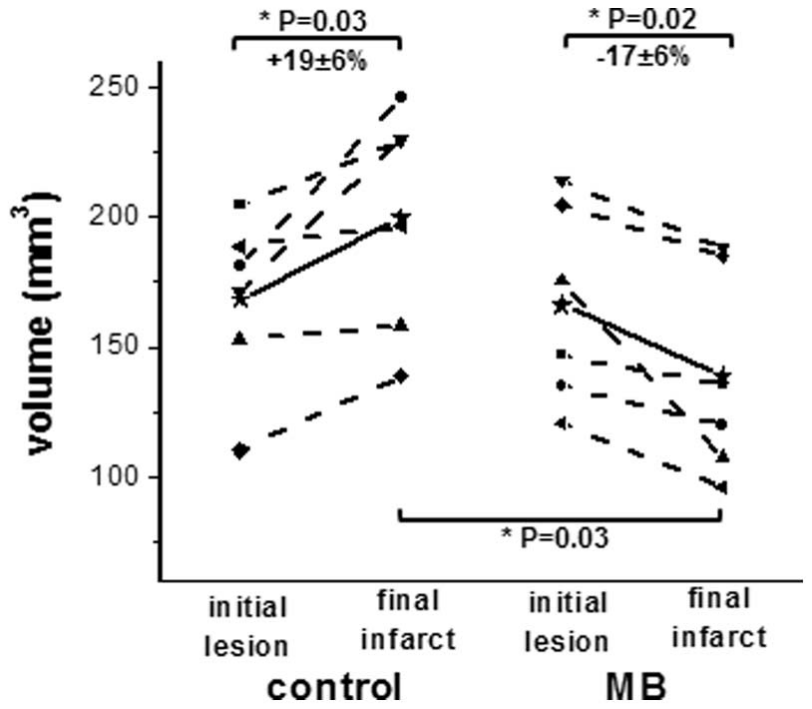
Initial lesion (30 mins) and final infarct (day 2) volumes of vehicle- and MB-treated rats subjected to 60 mins MCAO. Initial lesion and final infarct volumes of individual animal were connected using dot lines. Mean initial lesion and final infarct were connected using solid lines.

**Figure 4:**
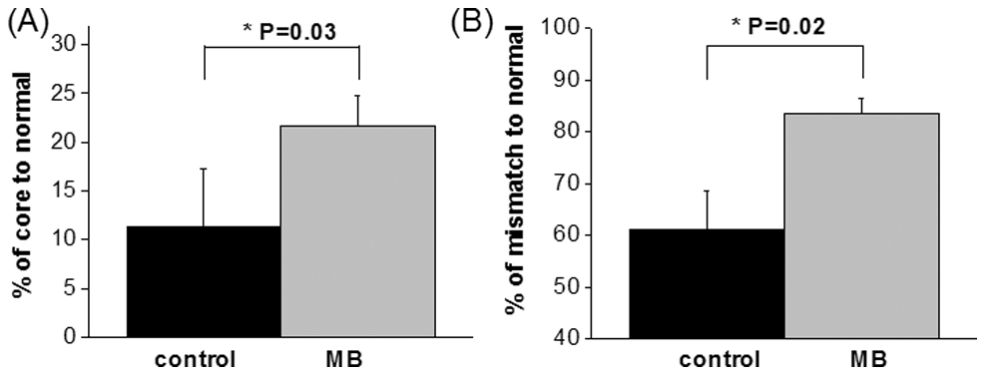
Percentage of survived core (C) and mismatch (D) tissues of control and MB-treated groups.

Representative ADC and CBF maps acquired at 30, 90, 120, and 180 minutes post-occlusion and on day-2 from control and MB-treated groups are shown in **Figure 2**. ISODATA clustering method was utilized to identify mismatch (yellow) and core (red) tissues based on the 30 min ADC and CBF maps. The core tissue volume was used as the initial lesion volume. Day-2 T2 maps, which offered the best contrast for determining final infarct volume, are shown at the bottom of **Figure 2**. The initial lesion volume at 30 mins and final T2-defined infarct volume 2 days after stroke were evaluated (**Figure 3**). The initial lesion volumes between vehicle- and MB-treated groups were not significantly different (P = 0.92). In the vehicle-treated group, the initial lesion grew larger by day 2 (19±6%, n = 6, P = 0.03). By contrast, in the MB-treated group, the initial lesion became smaller by day 2 (−17±5%, n = 6, P = 0.02). The difference in infarct volume on day 2 between vehicle and MB-treated groups was 30% (P = 0.03) (**Figure 3**). Tissue fate tracking analysis showed that MB salvaged more initial core pixels compared to controls (22±3% versus 11±3%, P = 0.03) (**Figure 4A**), and more mismatch pixels compared to controls (83±3% versus 61±8%, P = 0.02) (**Figure 4B**).

**Figure 5:**
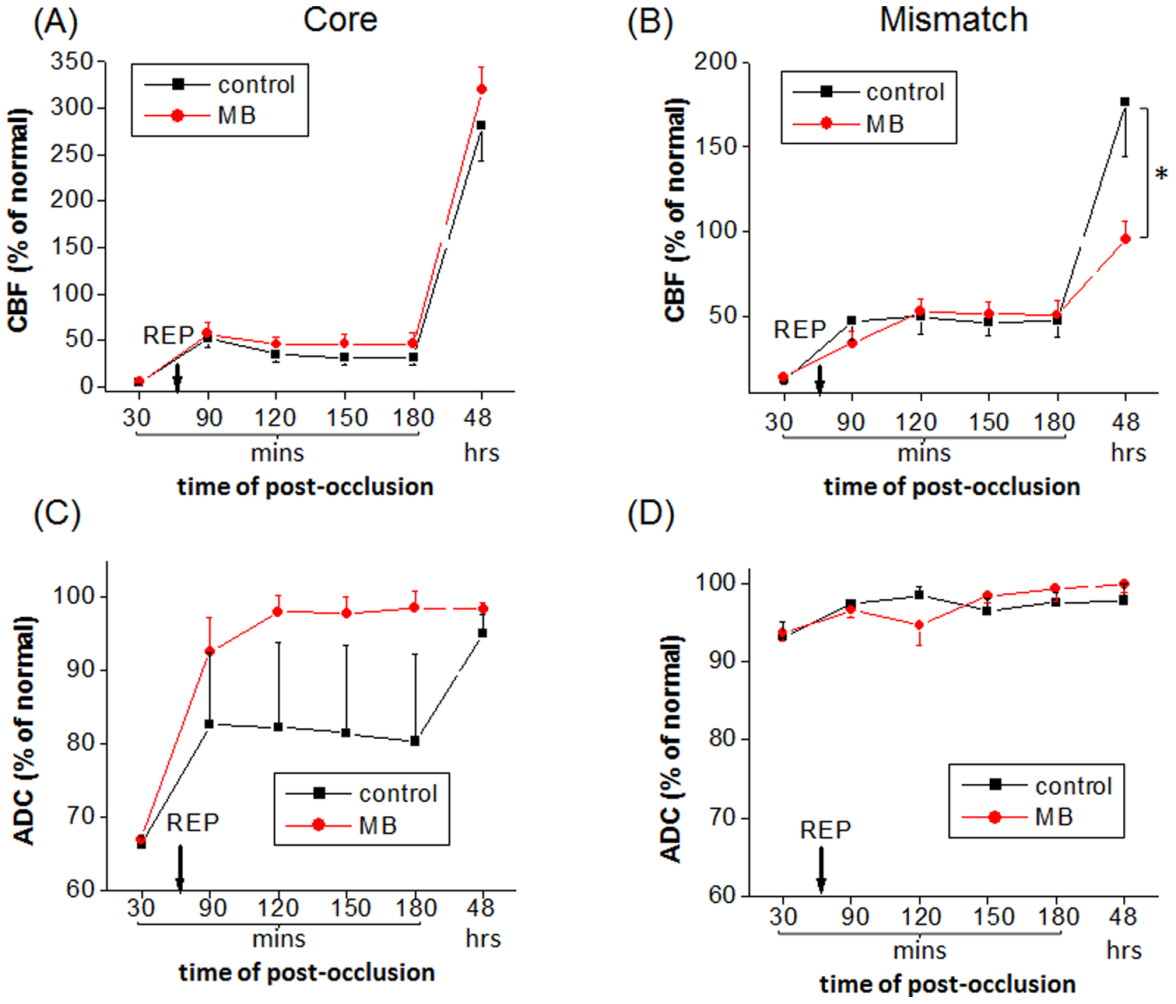
Normalized ADC and CBF values in acute phase and day-2 of core and mismatch tissues for control and MB-treated groups. Core and mismatch tissues were determined based on pre-reperfusion (30-min) data.

CBF and ADC values of core and mismatch tissues defined at 30 mins post-stroke were tracked over time for the MB and vehicle-treated groups (**Figure 5**). *Before* reperfusion, the ADC and CBF values of the core and mismatch tissues were not statistically different between the two groups. After reperfusion on day 0, the core tissue of MB-treated group showed slightly higher CBF and higher ADC values (i.e., toward normal value) than that of vehicle-treated group. On day-2, the core tissue of both groups showed similar hyperperfusion and pseudonormalized ADC. In other words, most of the initial lesion only transiently recovered, but was not salvaged.

After reperfusion on day 0, the mismatch's CBF and ADC values were not statistically different between the two groups. On day-2, mismatch tissue showed similar normal ADC values between the two groups, whereas the MB-treated group showed significantly lower hyperperfusion (P = 0.03) than that of the vehicle-treated group. On day-2, MB-treated group also showed significantly lower hyperperfusion than that of the vehicle-treated group.

**Figure 6:**
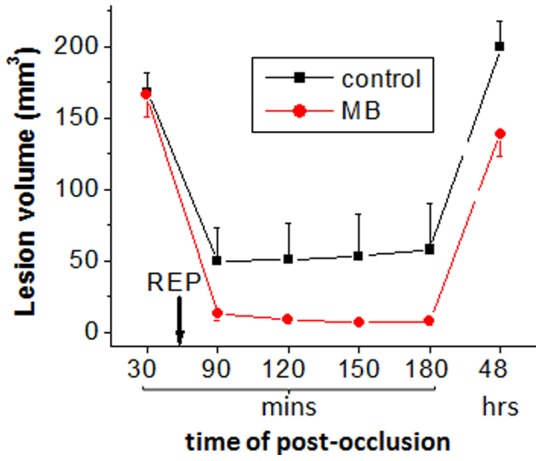
Evolution of the lesion volumes over time (determined using day-0 ADC maps and day-2 T2 maps) are demonstrated.

The lesion volumes determined using day-0 ADC maps and day-2 T2 maps (**Figure 6**) showed that much more tissue in MB-treated rats recovered (at least transiently) after reperfusion in day-0.

The mean NINDS stroke scores of MB versus vehicle-treated rats were, respectively, 1.5 versus 2 on day 2 (P = 0.04), and 1.0 versus 1.7 on day 7 (P = 0.01), indicative of general improvement in neurological status in the MB-treated group.

## Discussion

### MB distribution and dosing

MB has a low toxicity profile at low doses (0.5–1.0 mg/kg) and no negative side effects have been reported in animals and humans [2], [3] at low doses. However adverse effects (e.g., methemoglobinemia) have been reported at high doses (>10 mg/kg) [3], [12]. This difference is attributed to the hormetic behavior of MB resulting from increased oxidation rather than reduction at higher concentrations. MB reaches its maximum concentration in blood 5 mins after intravenous administration in humans [13]. MB rapidly accumulates in the brain, with concentrations 50 times higher in the brain than in the circulation one hour after intravenous administration in rats.^5^ The treatment protocol utilized in this study was based on published results that daily low-dose MB is safe in humans and similar dosing has been used in experimental models of Parkinson's disease [3], Alzheimer's disease [6], [7] and ischemic stroke [5]. Future studies will need to further optimize MRI dosing and timing regimens for treating ischemic stroke, and MRI offers a valuable means to longitudinally to evaluate efficacies.

### Physiological effects of MB

Although the mechanisms of MB action are well studied *in vitro*, the effects of MB on basal CBF, neurovascular coupling, evoked hemodynamic and oxygen consumption changes in the *in vivo* brain have only been recently reported. A single dose of MB (1 mg/kg i.v. over 20 mins) has substantial effects on cerebral metabolism, hemodynamics and evoked responses in *normal* rats. MB has also been shown to enhanced global glucose uptake, oxygen consumption and CBF [14]. MB markedly potentiated forepaw evoked BOLD, CBF and CMRO_2_ changes focally in the forepaw somatosensory cortices [15], suggesting that MB has focal effects that localized to regions with enhanced activity or metabolic stress. Moreover, during mild hypoxia (15% O_2_) MB-treated rats were better able to sustain global glucose uptake, oxygen consumption, and CBF compared to vehicle-treated rats [14]. MB-treated animals showed larger fMRI responses and O_2_ consumption changes compared to vehicle-treated rats [15]. MB did not have significant effects on blood gases, heart rate or fMRI responses following hypercapnia [15]. These findings further support the notion that MB is an energy enhancer *in vivo* under metabolically stressful conditions. Our findings of neuroprotection in cerebral ischemia are consistent with these unique MB properties.

### Lesion volumes

A randomized, double-blinded design was used to avoid bias. The stroke surgeon, the MRI operator, and the person who quantified lesion volumes did not know which animals received MB or vehicle treatment. Moreover, MRI was used to verify the presence of mismatch, to exclude incomplete occlusion, and ensure similar lesion sizes between the two groups at the 30-min time point. Such subject selection would not have been possible with terminal histological measurements. Recent clinical stroke trials have shown that patient selection is critical in order to maximize positive outcomes, and that any given treatment will unlikely be effective for all stroke patients.

Compared to controls, MB treatment decreased final infarct volume by 30%. To further identify which tissue types were salvaged, a novel feature of this study is that pixels were tracked on a pixel-by-pixel basis as a function of time. In vehicle-treated animals subjected to 60 min MCAO, only 11% of the initial ADC lesion defined at 30 mins post-stroke was salvaged by reperfusion. About 61% of the mismatch defined at 30 mins post-stroke was salvaged by reperfusion, consistent with the mismatch concept. By comparison, MB treatment salvaged substantially more of the initial ADC lesion (22%) and the initial mismatch (83%). The lesion volume changes are consistent with ADC changes that showed more tissue in MB-treated rats recovered after reperfusion in day-0 (**Figure 6**).

It was also observed that the MB-treated group showed significantly lower hyperperfusion than that of the vehicle-treated group. Hyperperfusion has been reported to be associated with poor outcome [16]. MB could also protect the blood-brain barrier [5], warranting further investigation. These findings are also consistent with the efficacy of MB in reducing lesion size, edema, and behavioral outcomes in traumatic brain injury (data not shown).

During ischemia, therapeutic approaches that buy time before recannulation (i.e., via sustaining energy (ATP) production in mitochondrial respiration) could help to minimize ischemic injury and extend the treatment time window. After recannulation it is also likely important to minimize reperfusion injury, such as from reactive oxygen species, which could further damage cells in the surrounding area. The unique energy-enhancing and antioxidant properties of MB likely target both neuroprotective mechanisms.

### Limitations and future directions

One limitation of this study is that although differences in neurological status were detected, the NINDS neurological score has a narrow dynamic range. Future studies will use more sensitive behavioral tests, such as the foot fault and limb asymmetry tests. While this study focused on acute stroke, we predict that MB could also have effects in functional recovery in chronic stroke. This is under investigation. MB treatment could also be combined with other therapies. Specifically, combining MB and normobaric oxygen treatment is being explored. Normobaric oxygen treatment is known to increase tissue oxygen, but it also increases reactive oxygen species. MB's antioxidant property may be used to curtail or minimize the increase in reactive oxygen species with normobaric oxygen treatment.

## Conclusions

This study employs imaging and analysis approaches to demonstrate that methylene blue (MB) significantly reduces infarct size and neurologic deficit in transient cerebral ischemia in rats. Such neuroprotective effects in ischemic stroke are consistent with the unique properties of MB as an energy-enhancer and an antioxidant. The novelties of this study included randomized double-blinded experimental design, longitudinal MRI tracking of ischemic tissue fate, and subject selection to ensure similar initial lesion volumes between groups using MRI. Because MB is already a FDA-grandfathered drug currently used in the clinics with an excellent safety profile at low dose, human stroke trials can be readily explored.
